# The role of laboratory indices on treatment response and survival in breast cancer receiving neoadjuvant chemotherapy

**DOI:** 10.1038/s41598-024-63096-7

**Published:** 2024-05-27

**Authors:** Sedat Yildirim, Akif Dogan, Goncagul Akdag, Zeynep Yüksel Yasar, Hamit Bal, Oguzcan Kinikoglu, Sila Oksuz, Ugur Ozkerim, Salih Tunbekici, Hacer Sahika Yildiz, Ozkan Alan, Sermin Coban Kokten, Deniz Isik, Heves Surmeli, Tugba Basoglu, Ozlem Nuray Sever, Hatice Odabas, Mahmut Emre Yildirim, Nedim Turan

**Affiliations:** 1 Department of Medical Oncology, Kartal Dr. Lütfi Kirdar City Hospital, Health Science University, Cevizli, D-100 Güney Yanyol, Cevizli Mevkii No:47, 34865 Kartal, Istanbul, Turkey; 2 Division of Medical Oncology, School of Medicine, Koç University, Istanbul, Turkey; 3 Department of Pathology, Kartal Dr. Lütfi Kirdar City Hospital, Health Science University, Istanbul, Turkey

**Keywords:** Early breast cancer, Neoadjuvant chemotherapy, Laboratory index, Pathologic complete response, Cancer, Chemical biology

## Abstract

Neoadjuvant chemotherapy (NACT) is the standard treatment for locally advanced, high-risk breast cancer. Pathological complete response (pCR) improves survival. Peripheral blood-derived indices reflecting systemic inflammation and nutritional status have long been used as predictive and prognostic markers in solid malignancies. This retrospective study investigates whether eight commonly used indices in patients receiving NACT affect pCR and survival. This study includes 624 locally advanced breast cancer patients who received NACT. The biomarker indices were calculated from peripheral blood samples taken two weeks before starting chemotherapy. The indices’ optimal cut-off values were determined using ROC Curve analysis. During a median follow-up period of 42 months, recurrence was detected in 146 patients, and 75 patients died. pCR was observed in 166 patients (26.6%). In univariate analysis, NLR, PLR, SII, PNI, HALP, and HRR were statistically significantly associated (*p* = 0.00; *p* = 0.03; *p* = 0.03; *p* = 0.02; *p* = 0.00; *p* = 0.02 respectively), but in multivariate analysis, only NLR was significantly predictive for pCR(*p* = 0.04). In multivariate analysis, the HGB/RDW score significantly predicted DFS(*p* = 0.04). The PNI score was identified as a marker predicting survival for both OS and PFS (*p* = 0.01, *p* = 0.01, respectively). In conclusion, peripheral blood-derived indices have prognostic and predictive values on pCR and survival. However, further studies are needed to validate our findings.

## Introduction

Breast cancer (BC) is the leading cause of cancer incidence and mortality among women^1^. With the expansion of screening programs and the increase in average life expectancy, more patients are diagnosed at early stages^2^. Despite promising clinical outcomes achieved through early diagnosis, advancements in surgical techniques, and multimodal treatments, breast cancer continues to be a leading cause of cancer-related deaths in women^3^. However, recurrence is still observed in many patients despite surgical resection, neoadjuvant, and adjuvant treatments^4^.

Neoadjuvant chemotherapy (NACT) is a significant treatment option for locally advanced breast cancer^5^. Pathological complete response (pCR) is associated with lower recurrence rates and indicates more favorable survival outcomes^6^.

The relationship between chronic inflammation and cancer is a popular topic in oncology. The value of inflammatory markers is being investigated both diagnostically and prognostically. Studies have shown inflammation contributes to tumor formation and progression^7–9^. Neutrophils, monocyte-derived macrophages, and platelets negatively impact the tumor microenvironment by promoting tumoral angiogenesis and tumor growth, thus having poor prognostic significance, whereas tumor-infiltrating lymphocytes indicate positive outcomes^10–13^.

Cancer-related anemia (CRA) can occur at any stage of cancer, from early to terminal, though it typically appears in advanced stages. This condition can arise secondary to chronic inflammation related to cancer, independent of antineoplastic therapy^14,15^. CRA has been shown to have a negative prognostic impact on disease-free survival (DFS) and overall survival (OS) in various types of cancer^16–18^. Red cell distribution width (RDW), reflecting changes in erythrocyte volume, has begun to be used as a prognostic marker in many malignancies^19,20^. Patients’ albumin levels, influenced by nutritional and inflammatory status, are adverse prognostic factors in various types of cancer^24,25^. Different laboratory indices derived from peripheral blood cells can provide information about the status of the intratumoral immune system^26–34^.

Our study aims to determine the predictive and prognostic values of commonly used indices when evaluated independently of their classical prognostic and predictive values and to assess whether they have any superiority. There is a lack of consistency in the results obtained from the commonly used indices across different studies. For this purpose, we selected and included eight widely used indices in the study, and we aimed to evaluate the predictive and prognostic value in complete response and survival in patients receiving neoadjuvant chemotherapy independently of classical prognostic factors.

## Materials and methods

All procedures performed in studies involving human participants were in accordance with the ethical standards of the institutional and/or national research committee and with the 1964 Helsinki Declaration and its later amendments or comparable ethical standards. Ethics/Institutional Review Board approval of research with the number 2023/514/246/27, dated 29.03.2023.

**Figure 1:**
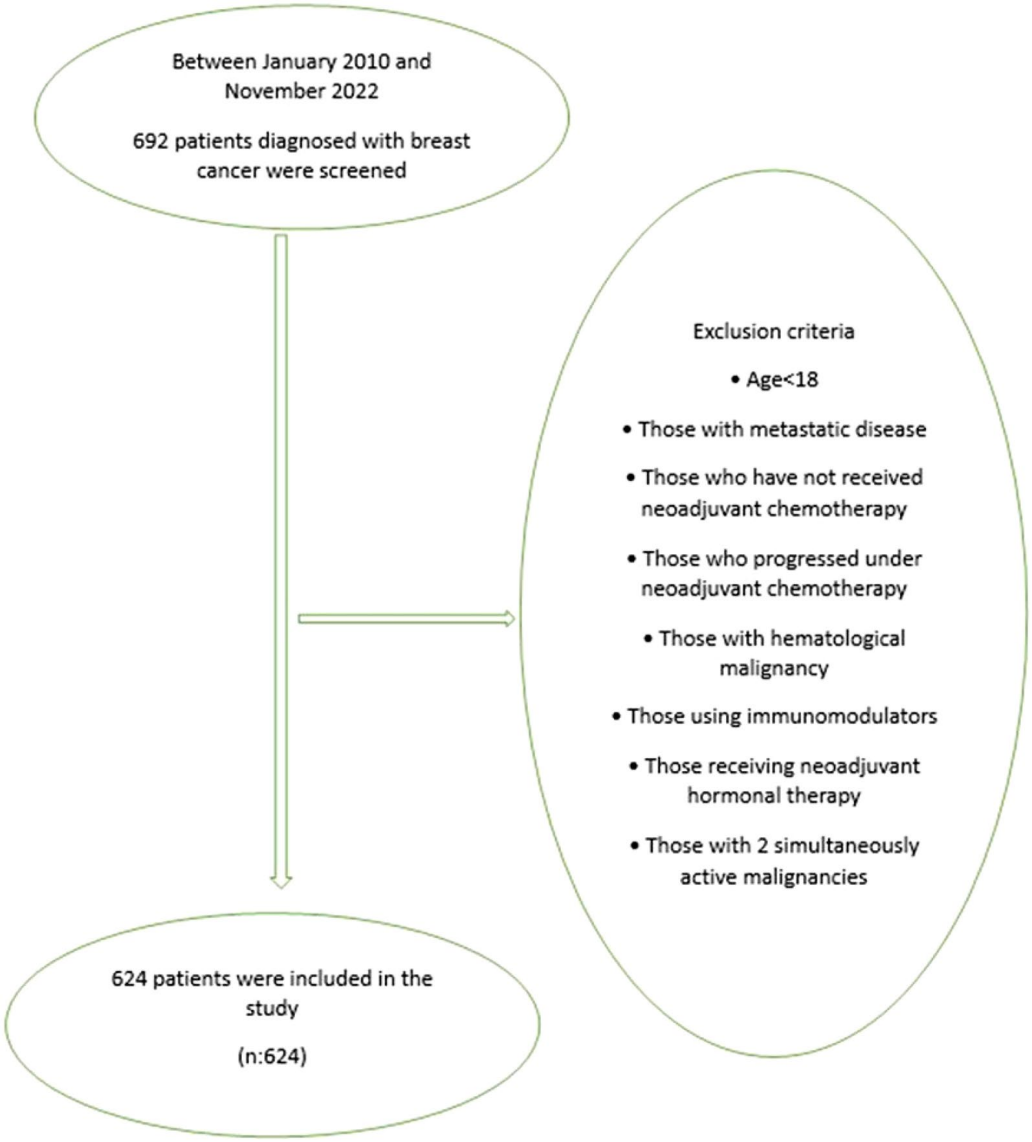
Patient selection method in our study.

Our study retrospectively reviewed the oncological records of 692 patients diagnosed with breast cancer between January 2010 and November 2022, who underwent pathological evaluation after NACT and had complete follow-up files in our clinic. Exclusions are shown in Fig. 1.

Patients’ medical records were reviewed for demographic characteristics (Table 1), histological features (Table 2), pathological data (Table 3), and laboratory results. Immunohistochemical staining from diagnostic biopsies was used to classify patients with > 1% expression levels of ER or PR as hormone positive. HER-2 status was determined as positive in patients with a score of 3 or a suspiciously positive score of 2 with positive FISH results. Patients with < 1% ER and PR expression levels and concurrently negative for HER-2 were classified as triple-negative. The response to neoadjuvant chemotherapy was assessed through pathology reports of surgery. Staging was based on the American Joint Committee on Cancer (AJCC) Staging Manual, eighth edition. Complete blood counts and biochemical values were reviewed before starting NACT. NLR, MLR, HRR, PLR, SII, HALP, PNI, and PIV were calculated using absolute neutrophil, lymphocyte, monocyte, platelet counts, hemoglobin, albumin, and red cell distribution width values. These calculations were made as follows:

- NLR = neutrophil count (10^3^/mm^3^)/lymphocyte count (10^3^/mm^3^)
- MLR = monocyte count (10^3^/mm^3^)/lymphocyte count (10^3^/mm^3^)
- PLR = platelet count (10^3^/uL)/lymphocyte count (10^3^/mm^3^)
- SII = platelet count (10^3^/uL) × neutrophil count (10^3^/mm^3^)/lymphocyte count (10^3^/mm^3^)
- PNI = serum albumin (g/L) + 5 × lymphocyte count (10^3^/mm^3^)
- HALP = hemoglobin (g/dL) × albumin (g/L) × lymphocyte count (10^3^/mm^3^)/platelets (10^3^/uL)
- PIV = neutrophil count (10^3^/mm^3^) × platelet count (10^3^/uL) × monocyte count (10^3^/mm^3^)/lymphocyte count (10^3^/mm^3^)
- HRR = hemoglobin (g/dL)/RDW %

**Table 1:** Demographic and clinical characteristics of patients and tumor.

Variables	N (624)	(%)
Age (years)		
≤ 50	331	53.0
> 50	293	47.0
Gender		
Female	621	99.5
Male	3	0.5
Menopausal status		
Premenopausal	330	52.9
Postmenopausal	290	46.5
Missing	4	0.6
Chemotherapy regimens		
Anthracycline + Taxane	581	93.1
Anthracycline-based	23	3.7
Taxane-based	20	3.2
T stage		
T1–T2	506	81.1
T3–T4	97	16.2
Missing	21	2.7
N stage		
N0	50	8.0
N1	342	54.8
N2	144	23.1
N3	66	10.6
Missing	22	3.5
Histological type		
Infiltrating carcinoma (NOS)	376	60.3
Invasive lobular carcinoma	30	4.8
Invasive ductal carcinoma	199	31.9
Other*	19	3.0
Patient subgroups		
Hormone positive and Her-2** negative	293	46.2
Her-2 positive	244	39.9
Triple negative	87	13.9
Ki-67 score		
≤ 20	152	24.4
> 20	383	61.4
Missing	89	14.3
Histological grade		
Grade < 3	382	61.2
Grade ≥ 3	220	35.3
Unknown	22	3.5

*Others: tubular carcinoma, medullary carcinoma and mucinous carcinoma,

**HER2: human epidermal growth factor receptor 2,

**Table 2:** Univariate and multivariate logistic regression results of various index variables on complete response.

		Univariate		Multivariate	
Indıces	Cut-off	OR (95% CI)	*p*	OR (95% CI)	*p*
NLR	≤ 2.25	1.88 (1.24–2.84)	0.003*	1.77 (1.02–3,05)	0.040*
MLR	≤ 0.25	1.28 (0.84–1,94)	0.244		
PLR	≤ 141.35	1.55 (1.03–2,33)	0.035*	0.90 (0.43–1,84)	0.773
SII	≤ 639.66	1.54 (1.02–2,31)	0.039*	0.82 (0.46–1,47)	0.525
PIV	≤ 300.09	1.08 (0.72–1.62)	0.703		
PNI	≥ 53.97	1.57 (1.04–2,37)	0.029*	1.06 (0.65–1,72)	0.799
HALP	≥ 38.72	1.87 (1.24–2.83)	0.003*	1.67 (0.78–3.59)	0.185
HRR	≥ 0.92	1.63 (1.07–2.47)	0.020*	1.36 (0.87–2,12)	0.172

*OR* Odds ratio, *CI* Confidence ınterval, *NLR* Neutrophil-to-lymphocyte ratio, *MLR* Monocyte-to-lymphocyte ratio, *PLR* Platelet-to-lymphocyte ratio, *SII* Systemic ımmune-ınflammation ındex, *PIV* Platelet-ımmune-ınflammation ındex, *PNI* Prognostic nutritional ındex, *HALP* Hemoglobin-albumin-lymphocyte-platelet ındex, *HRR* Hemoglobin-to-red cell distribution width ratio.

*A *p*-value < 0.05 was considered statistically significant.

**Table 3:** Multivariate cox regression results of various index variables on OS and PFS.

		OS		PFS	
		Multivariate		Multivariate	
Variables	Cut-off	HR (95% CI)	*p*	HR (95% CI)	*p*
NLR	≥ 2.25	0.78 (0.41–1.46)	0.440	1.01 (0.64–1,60)	0.953
MLR	≥ 0.25	1.14 (0.62–2,08)	0.659	1.31 (0.86–2,06)	0.206
PLR	≥ 148.1	1.55 (0.25–1,27)	0.173	0.68 (0.27–1,25)	0.223
SSI	≥ 670.72	1.54 (0.72–3,25)	0.263	1.41 (0.82–2,41)	0.205
PIV	≥ 336.3	1.08 (0.67–2.75)	0.386	0.95 (0.58–1,55)	0.857
PNI	≤ 52.67	1.57 (1.16–3,85)	0.014*	1.63 (1.09–2,42)	0.016*
HALP	≤ 36.2	1.87 (0.89–5.10)	0.089	1.29 (0.68–2.43)	0.423
HRR	≤ 0.90	1.63 (0.74–2.02)	0.428	1.42 (1.01–2,03)	0.047*

*HR* Hazard ratio, *CI* Confidence ınterval, *NLR* Neutrophil-to-lymphocyte ratio, *MLR* Monocyte-to-lymphocyte ratio, *PLR* Platelet-to-lymphocyte ratio, *SSI* Systemic ımmune-ınflammation ındex, *PIV* Platelet-ımmune-ınflammation ındex, *PNI* prognostic nutritional ındex, *HALP* Hemoglobin-albumin-lymphocyte-platelet ındex, *HRR* Hemoglobin-to-red cell distribution width ratio.

*A *p*-value < 0.05 was considered statistically significant.

pCR was defined as the absence of tumor cells in pathology samples from mastectomy or excised tumors and lymph nodes following neoadjuvant chemotherapy. DFS was calculated as the time (in months) from curative surgery to recurrence and/or death (whichever occurred first). OS was calculated as the time (in months) from diagnosis to death or the last check-up time for patients who did not die.

Statistical analyses were performed using IBM SPSS Statistics for Windows, Version 25.0 (Statistical Package for the Social Sciences, IBM Corp., Armonk, NY, USA). Descriptive statistics were presented as n and % for categorical variables and mean ± SD or median (min–max) for continuous variables. ROC Curve analysis results were provided for the investigated NLR, MLR, HRR, PLR, SII, HALP, PNI, and PIV indices’ ability to predict mortality. Kaplan Meier method was used to compare OS and DFS durations among various clinical parameter groups. Univariate and Multivariate Logistic Regression results were provided for the risk of pCR on various clinical factors. Finally, Multivariate Cox regression results were provided for the impact of different clinical variables on OS and DFS. A *p*-value of < 0.05 was considered statistically significant.

## Consent to participate

Patient data were obtained retrospectively from patient records after obtaining written informed consent from the patients or their relatives.

## Results

Of the 692 patients screened, 624 met the inclusion and exclusion criteria and were included in the study. The average age of the patients was 50 years (range 22–82 years), with three (0.5%) being male. There were 330 premenopausal patients (52.9%). The general characteristics of the patients and tumors are presented in Table 1.

pCR was observed in 166 patients (26.6%) following NACT. Among the 293 hormone-positive patients, complete response was seen in 22(7.5%), partial response in 191(65.1%), and no response to NACT in 80(27.3%) patients. The highest pCR rate was observed in HER2-positive disease (47.9%), with complete response in 117 (47.9%) of 244 HER-2-positive patients. Among 87 triple-negative patients, a complete response was seen in 27 (31.0%). Laboratory values were calculated according to NLR, MLR, PLR, SII, PIV, PNI, HALP, and HRR indices, and cut-off values for pCR were determined using ROC Curve analysis (Table 2). These values were examined along with their sensitivity and specificity. While Univariate analysis found NLR, PLR, SII, PNI, HALP, and HRR (*p* = 0.00; *p* = 0.03; *p* = 0.03; *p* = 0.02; *p* = 0.00; *p* = 0.02, respectively) to be statistically significantly associated, only the NLR index was found to be statistically significant in predicting pCR in Multivariate analysis (*p* = 0.04).

**Figure 2:**
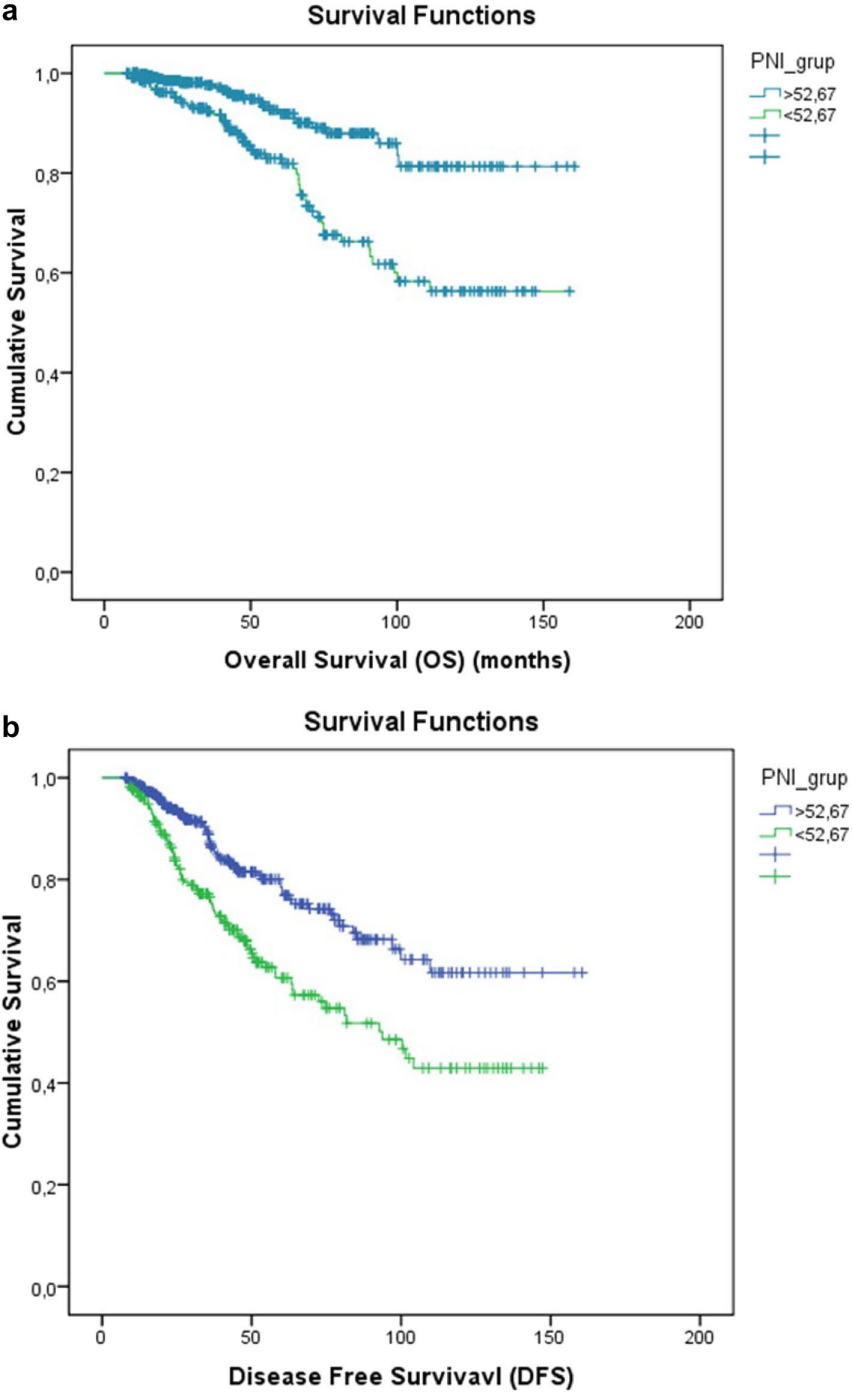
(**a**) Kaplan Meier graphs of overall survival according to groups with high and low Prognostic Nutritional Index (PNI) values. (**b**) Kaplan Meier graphs of disease-free survival according to groups with high and low Prognostic Nutritional Index (PNI) values.

The median follow-up period was 42 months, during which 75 (12%) patients died, and recurrence was observed in 146 (23.3%) patients. Laboratory values were calculated according to NLR, MLR, PLR, SII, PIV, PNI, HALP, and HRR indices with determined cut-off values for DFS and OS (Table 3). While Univariate analysis found significance in all indices, Multivariate analysis observed that the HGB/RDW score statistically significantly predicted DFS (*p* = 0.04). It was found that the NLR (neutrophil-to-lymphocyte ratio) was not a good predictor for DFS (disease-free survival) and OS (overall survival) in hormone-positive and triple-negative subtypes of breast cancer (*p* = 0.250, *p* = 0.087, *p* = 0.698, and *p* = 0.389 respectively). However, in the HER2-positive subtype, it was not predictive for DFS (*p* = 0.073) but found to be significantly prognostic for OS (*p* = 0.032). In the entire patient group, high NLR was observed to be a poor prognostic factor for DFS and OS in univariate analysis (*p* = 0.004 and *p* = 0.027, respectively). However, in multivariate analysis, this significance was not observed (*p* = 0.953 and *p* = 0.440, respectively). The PNI score was identified as a marker predicting survival for both OS and PFS (*p* = 0.01, *p* = 0.01, respectively) (Fig. 2a and b).

## Discussion

In our study, we explored the relationship between laboratory indices and survival outcomes, DFS, and pCR in breast cancer patients undergoing NACT. Our approach was unique because we simultaneously analyzed multiple indices in a selected patient group to establish the relationship between survival and pCR. We found that the NLR index predicted pCR. While HRR PNI was predictive for DFS, only PNI significantly correlated with overall survival (OS). Our study stands out in the literature for investigating the relationship of 8 commonly used and long-established indices with pCR and DFS/OS in the same patient cohort.

Clinical studies have been conducted to analyze the indices derived from laboratory values. However, these studies have reported conflicting findings with respect to different indices and even for the same index across various studies. The superiority of different indices also remains debatable, with contradictory results reported in various studies^35–39^. For example, one study reported NLR to be predictive of neoadjuvant response and an independent prognostic marker for survival^39^. On the other hand, another study demonstrated that NLR was not predictive of neoadjuvant response and was not an independent prognostic marker for survival^36^. These inconsistencies suggest that laboratory-derived indices may not be reliable prognostic and predictive biomarkers in breast cancer, particularly when evaluated independently of established prognostic and predictive markers like lymph node status and hormone status.

Inflammation plays a crucial role in all stages of carcinogenesis and progression, as well as in the ineffectiveness of anti-cancer treatments^40–42^. Numerous studies have shown that systemic inflammation in cancer patients is associated with poorer survival, thereby motivating the clinical use of inflammatory markers as prognostic indicators^43,44^.

As the first line of defense, the acute inflammatory response represents innate and adaptive immune responses against any damage or foreign entities perceived by the body, such as infections. Most infiltrating inflammatory cells in acute inflammation are neutrophils^45,46^. While acute inflammation contributes to cancer cell death by inducing an anti-tumor immune response, chronic inflammation caused by treatment promotes therapeutic resistance and cancer progression^47^.

A meta-analysis by Zhou et al., encompassing 17 clinical studies and 5504 patients, demonstrated that a lower NLR value significantly predicted both pCR and improved DFS and OS^43^. According to a meta-analysis by Cullinane et al., which involved eight clinical studies and 1586 patients, a low NLR value was found to be consistent with better PCR. However, the results did not reach statistical significance in terms of improved 5-year DFS, despite the fact that patients with lower NLR levels showed some improvement^48^. In another meta-analysis by Xue et al., a higher NLR value was associated with worse pathological complete response, but the prognostic value of NLR for DFS and OS could not be demonstrated^49^. On the other hand, a study by Feeney G. found that low preoperative NLR was predictive of complete response^50^. However, some studies have proven the opposite. For example, in two different studies, no significant predictive relationship was observed between NLR level and complete response. Additionally, both studies observed that the NLR value was not an independent prognostic index for DFS^36,37^. In our study, an NLR value below 2.25 was significantly associated with good pCR in univariate and multivariate analyses. But it was not predictive of PFS and OS. There are conflicting findings in studies regarding the NLR index. While some studies suggest that it is useful for predicting complete response, disease-free survival (DFS), and overall survival (OS), it is only available in studies that show its predictive ability for complete response or survival. However, other studies are showing that it is not predictive of complete response, DFS and OS. The reliability of these indices obtained from peripheral blood is questionable. The use of these indices should be considered independently of clinicopathological features. If these indices are to be used, the patient’s inflammation and nutritional status should be considered. Given that NLR primarily reflects acute inflammation, it is associated with pCR, an early-stage malignancy condition, but not with long-term indicators such as DFS and OS.

Chronic inflammation is implicated in every step of tumor formation, including malignant cellular transformation, proliferation, invasion, angiogenesis, and metastasis^51^. It plays a central role in the emergence and progression of tumors and contributes to resistance to chemotherapy and radiotherapy^40,41^. Chronic inflammation is characterized by simultaneous tissue destruction and healing, with macrophages and lymphocytes as the primary immune cells infiltrating chronic inflammation areas, also playing a role in forming immunological memory^45^.

Malnutrition can affect the progression and survival of cancer patients. Several studies have linked malnutrition in cancer to poor prognosis, reduced quality of life (QoL), and lower activity levels, as well as increased adverse symptoms related to treatment and reduced tumor response to therapy^52,53^. Serum albumin levels are closely associated with malnutrition and are independent predictors of inflammation and mortality^21^.

In a study by Mantzorou et al., which included breast cancer patients, PNI was found to be predictive for disease progression and prognosis^52^. Similarly, a study by Chen et al. observed that a high PNI value was an independent significant prognostic index for better DFS and OS in breast cancer patients receiving NACT^25^. Another study involving over 1100 patients found that pre-treatment PNI was a reliable predictor of pCR in breast cancer patients^54^. However, in the study by Yang et al., it was observed that pCR was not a predictive marker in breast cancer patients receiving PNI and NACT^55^. Lastly, a study by Oba T. divided patients receiving NACT into two groups based on their PNI before treatment. There was no difference in DFS between the high PNI and low PNI groups^56^. In our study, however, high PNI values did not correlate with pCR but showed a close correlation with better DFS and OS. The limitations present in NLR are also present in PNI, and as such, peripheral indices should be considered in conjunction with clinicopathological characteristics. As mentioned earlier, lymphocytes are more prominent in chronic inflammation, and albumin levels are also at the forefront in malnutrition and chronic inflammation. Therefore, the effects of PNI, calculated using albumin and lymphocyte counts, emerge in the longer term rather than in determining the immediate pCR after NACT.

Although studies support the predictive value of indices calculated from peripheral blood values, it is unclear which index possesses the most accurate predictive power. The literature presents conflicting publications on this matter^8,29,43^.

Our study’s limitations include its retrospective nature and being a single-center experience. However, its strengths lie in having a large patient cohort and investigating eight commonly used indices for pCR and DFS/OS relationships in the same group.

## Conclusions

There are conflicting results regarding the indices commonly used today. When using these indices, it is important to consider the clinicopathologic and demographic aspects of the patient, especially those with breast cancer. These indices, which are readily available, cost-effective, and acceptable, might assist oncologists in choosing relevant therapies for patients, it is crucial to persist in their development. Nevertheless, it is important to consider that the indices obtained from peripheral blood are significantly affected by both the patient’s inflammatory and nutritional condition. Our study identified a statistically significant relationship between pre-treatment lower NLR and pCR, which is closely associated with acute inflammation. We also demonstrated a statistically significant relation between high PNI, DFS, and OS, reflecting its close association with nutritional status and chronic inflammation. However, prospective randomized studies with larger patient populations across multiple centers are necessary to generalize these results.

## Data Availability

The datasets generated during and/or analyzed during the current study are not publicly available but are available from the corresponding author on reasonable request.
